# Polydioxanone Internal Support Matrix: A Rationale for Prophylactic Internal Bra Support in Breast Augmentation

**DOI:** 10.1093/asjof/ojac021

**Published:** 2022-03-30

**Authors:** Julia A Chiemi, S Sean Kelishadi

## Abstract

**Background:**

Textured breast implants have been used in aesthetic breast surgery to decrease the rates of implant malposition. A recent analysis of a large-volume single-surgeon experience found statistically similar rates of malposition in smooth vs micro-textured breast implants.

**Objectives:**

Prophylactic use of a polydioxanone (PDO) internal support matrix in breast augmentation was hypothesized to prevent scar malposition and increase pocket control.

**Methods:**

In total, 200 patients received silicone gel primary augmentations performed by a single surgeon from January 2018 to December 2020; 84 patients received smooth implants alone; 49 patients received micro-textured implants; and 67 patients received smooth implants plus PDO internal support matrix. All surgeries were performed in the dual plane using an inframammary incision. Implant-related complications and scar malposition were recorded and compared.

**Results:**

No significant difference in implant-related complication rates was found between shell types (3.57% for smooth devices alone and 2.04% for textured devices [*P* = 0.62; 95% CI −0.06 to 0.01]). There were zero complications in the smooth plus mesh study arm. A comparison of scar malposition rates between the smooth alone and textured groups revealed no significant difference (15.4% for smooth devices and 8.16% for textured devices [*P* = 0.23; 95% CI −0.12 to 0.01]). The smooth implant group with the prophylactic placement of PDO mesh had the lowest scar malposition rate of 4.48%, a significant difference compared with the smooth devices alone (*P* = 0.03; 95% CI −0.21 to −0.01).

**Conclusions:**

Micro-textured devices show a trend toward decreased scar malposition, although not significant. Prophylactic use of PDO internal support matrix in silicone gel breast augmentation is safe and has the lowest incidence of scar malposition.

**Level of Evidence: 3:**

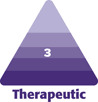

Textured breast implants have been used in breast surgery since 1968 to decrease the rates of implant malposition.^1^ The higher coefficient of friction associated with textured shells has been advertised to provide a protective effect against implant rotation and movement due to higher rates of tissue incorporation^2^ as well as a lower risk of capsular contracture compared with smooth breast implants.^3^ In recent years, new concerns regarding the long-term safety of textured implants have emerged as epidemiological knowledge on breast implant-associated anaplastic large cell lymphoma (BIA-ALCL) has developed. BIA-ALCL is a novel classification of a rare breast implant-associated strain of T-cell lymphoma that has been associated with textured breast implants^2,4,5^—its pathogenesis is unclear, but current theories focus on Gram-negative bacteria proliferation and lymphocyte stimulation.^6^ These concerns around the safety of textured implants were further amplified by the 2019 FDA trials and the subsequent recall of Allergan’s Biocell macro-textured breast implants (Irvine, CA) in the United States,^7^ leading many physicians to discontinue or reconsider their use of textured implants in aesthetic breast surgery.

A recent analysis of a large-volume single-surgeon experience found statistically similar incidences of scar migration in smooth vs micro-textured breast implants,^8^ suggesting that micro-textured implants may not be fully protective against implant malposition as advertised (Table 1). However, a viable alternative to texture is necessary should these implant shells be phased out of the US market, especially for use in patients with anatomical features (such as pectus carinatum, pectus excavatum, or a mobile vs manipulated inframammary fold [IMF] that creates fold instability) that put them at risk to developing malposition with smooth implants. Malposition is defined as the unsatisfactory location of an implant on the breast mound, often leading to a poor aesthetic outcome. Though malposition can be an issue from the outset after breast augmentation when pocket control is poorly established, it is also a problem that develops over time throughout the healing process. A common marker of implant malposition is characterized by scar movement to an unfavorable position during or following the course of implants settling after breast augmentation. The vertical migration of the scar, even by a few millimeters, can result in asymmetry, greater scar visibility, and poor shape for which patients may seek reoperation to correct. Even minor “malposition” can be of great concern to some patients, but not to others; the expert surgeon’s desire for greater precision and predictability leads us to seek out options for even greater control than what is considered the norm. Prophylactic use of a polydioxanone (PDO) internal support matrix in cosmetic breast augmentation to prevent scar migration as a marker of malposition has never before been described.

**Table 1.** Scaled Prevalence Rates of Implant Malposition Recorded Between the Smooth and Micro-Textured Implant Groups Throughout the Duration of Follow-up

Implant type	Prevalence by cohort	*P*-value
Smooth implants (n = 84)	15.4%	0.226156
Micro-textured implants (n = 49)	8.16%	
Total	12.7%	

Data from Chiemi and Kelishadi.^8^

Internal support matrices (hereinafter referred to as “mesh”) have been previously described in breast surgery for reinforcement of soft tissue and are available in a variety of materials.^9,10^ DuraSorb (SIA, Chicago, IL), a PDO mesh, was cleared by the FDA in 2018 for soft tissue support.^11^ Its early tissue integration and absorption profile lend it to the investigation as an option for decreasing scar migration and providing durable long-term stability with the ultimate pocket control in aesthetic breast augmentation.^12,13^

## METHODS

A retrospective cohort analysis was conducted using data collected from 200 consecutive primary augmentation mammaplasty cases performed between January 2018 and July 2021. All surgeries were performed by the senior author (S.S.K.). A total of 200 surgeries were performed with bilateral silicone gel breast implants, of which 84 patients received smooth silicone gel breast implants alone; 49 patients received micro-textured silicone gel breast implants (all micro-textured implants used were Sientra (Santa Barbara, CA) Opus Luxe implants, with the exception of one case in which the patient received Mentor (Irvine, CA) Siltex implants due to a sizing preference); and 67 patients received smooth silicone gel breast implants plus PDO internal support matrix (Table 2). The patients in all 3 groups were similarly healthy, and the average implant size used across groups was similar. All patients were female, ranging from ages 18 to 64 years (the average patient age in this study was 30 years). 

**Table 2.** Treatment Group Proportions and Sizes

Implant type	Primary augmentation-mammaplasty (n = 200)
Smooth implants alone	42.0% (84)
Micro-textured implants alone	24.5% (49)
Smooth implants + PDO mesh	33.5% (67)

PDO, polydioxanone.

Written consent was provided, by which the patients agreed to the use and analysis of their data. Patients in the micro-textured and mesh cohorts were given additional counseling during their consultation and/or preoperative appointment on the details of their treatment plan and how it compares to using smooth-shelled implants alone in breast augmentation. During counseling, patients in the mesh cohort were informed of the investigational nature of their treatment, and that the mesh being used is FDA approved but has not been specifically labeled for use against an implant.

In all breast implant cases, techniques^14^ were employed with the aim of reducing the risk of bacterial contamination and/or capsular contracture during surgery. Intravenous antibiotics were administered to patients at the start of the anesthetic (2 g of IV Cefazolin except where patients indicated an antibiotic allergy). All patients in the study received an inframammary incision for dual-plane breast augmentation along with nipple shields. Careful atraumatic dissection with electrocautery was performed to have a bloodless field. Pocket irrigation was performed in all cases, with the preferred irrigation being a triple antibiotic solution containing Cefazolin, Bacitracin, and Gentamicin, except when patients had an allergy or if supplies of ingredients for the triple antibiotic were unavailable; in those situations, PhaseOne (Nashville, TN) hypochlorous acid (HOCl) or 50% betadine solution was used. The use of an introduction sleeve, new gloves before handling the implants, and careful attention to sterile technique were all employed to minimize the bacterial burden. Three-layered suture closure was used for all cases. Drains were never used. Postoperative antibiotic prophylaxis was employed, with all patients receiving a cephalosporin antibiotic (Cephalexin 500 mg, PO TID) for 10 days except in the case of a known allergy.

For patients receiving implants alone (both smooth and micro-textured), 3-layered suture closure was initiated after implant insertion using an introduction sleeve. For patients receiving smooth implants with PDO mesh, implants were inserted using the same no-touch technique described above. After removal from sterile packaging, the 10 × 25 cm mesh was soaked in the same preferred pocket irrigation solution as described above and was cut in half. Each half was used to stabilize the respective breast pocket from the entire medial to lateral border of the IMF along its most inferior edge and cover at least half the height of the breast implant along its superior border. The mesh was inset with deep stitches to the Scarpa’s fascia and sometimes the periosteum of the rib using 2-0 Vicryl suture along its inferior edge spanning as far medial and lateral as could be reached along the IMF incision; most of the patients received 3 or 4 interrupted sutures approximately 1 cm apart. The mesh was subsequently unfurled, making sure that the smooth surface of the matrix faced the implant and its rough surface faced the breast tissue. Three-layered suture closure was then performed per the usual routine: breast fascia to Scarpa’s fascia (2-0 Vicryl), deep dermis (3-0 PDS), and subcuticular layer (4-0 Monocryl).

**Figure 1.**
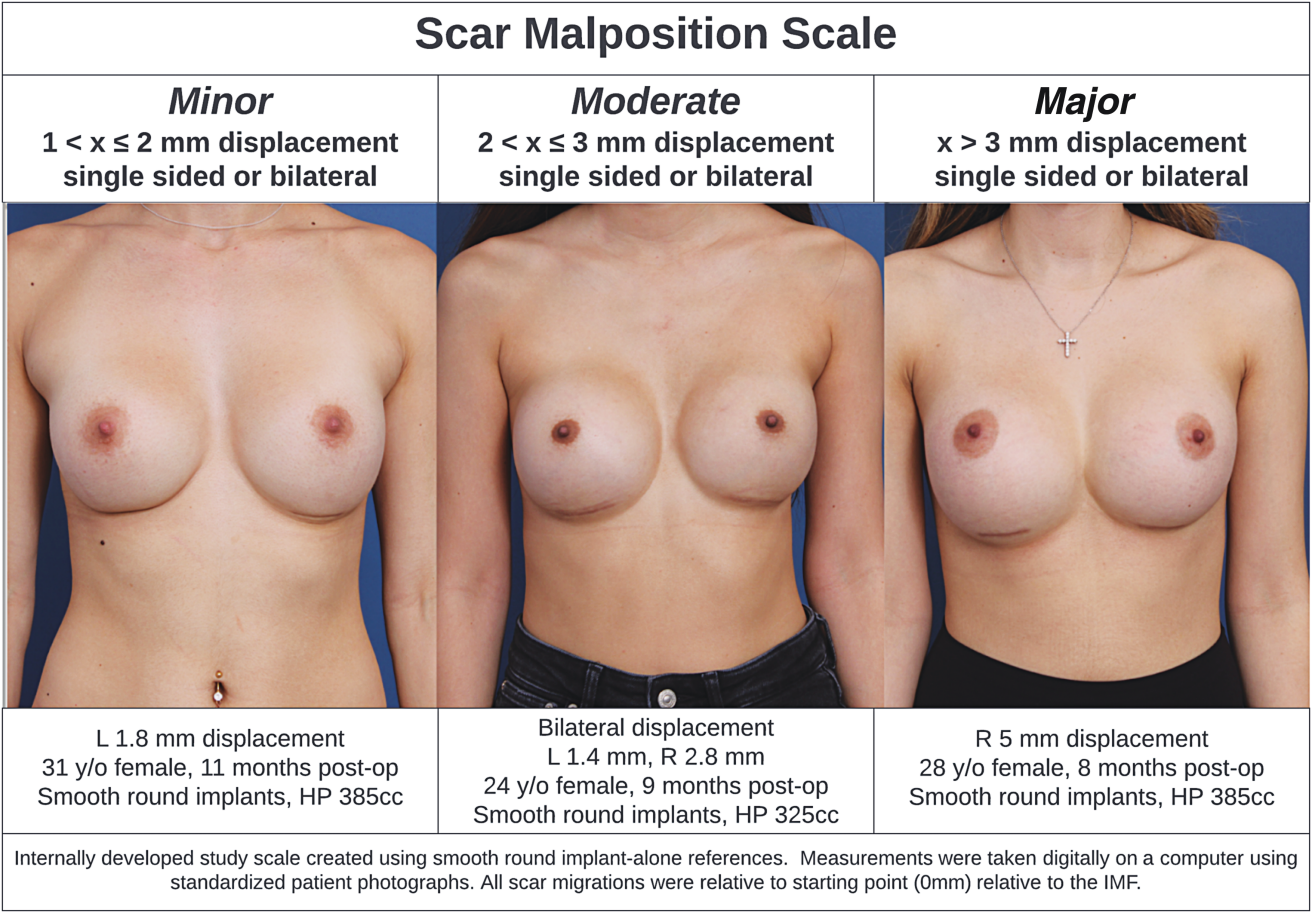
Study scar malposition classification scale based on scar movement from original inframammary fold (IMF) placement in millimeters. For all 3 patients shown, the starting scar position after surgery was directly in the IMF (0 mm migration). HP, high profile.

Patients were monitored with the typical in-person follow-up schedule of 1 week postsurgery, 1 month postsurgery, 3 months postsurgery, 6 months postsurgery, and yearly follow-up appointments for each subsequent anniversary. Patient photographs were reviewed at minimum 2 months follow-up and beyond, with the latest available patient data beyond 2 months utilized for this study. Complications related to breast implants including skin infection, wound dehiscence, hematoma, seroma, capsular contracture (Baker grade III or IV), and need for reoperation were recorded. Implant migration was also gauged by comparing scar position in postoperative appointment photographs with longitudinal follow-up photographs ranging from 2 months to 2 years postsurgery. Scar malposition was classified into 3 categories (Figure 1): minor (1 < x ≤ 2 mm, single-side or bilateral), moderate (2 < x ≤ 3 mm, single-side or bilateral), and major (x > 3 mm, single-side or bilateral). Statistical analyses to compare the complication and scar malposition rates of the smooth alone, micro-textured alone, and smooth implants with PDO mesh were performed using unpaired 2-tailed *t*-tests, and 95% CIs were constructed.

## RESULTS

The mean follow-up time period was 9.3 months (SD 1.23) for the smooth devices-alone cohort, 8.6 months (SD 1.04) for the micro-textured devices cohort, and 9.5 months (SD 1.12) for the smooth devices plus PDO mesh cohort. No significant difference in the prevalence of implant-related complications was found between implant shell types. Smooth devices alone had a complication rate of 3.57% (3 total patients—2 hematomas and 1 implant extrusion). Micro-textured devices had a complication rate of 2.04% (1 implant extrusion) [*P-*value 0.62; 95% CI −0.06 to 0.01]. There were zero complications in the smooth implant plus mesh study arm. Comparison of the scar malposition rates between the smooth alone and micro-textured implant groups also revealed no statistically significant difference (15.4% for smooth devices and 8.16% for micro-textured devices [*P*-value 0.23; 95% CI −0.12 to 0.01]). (Table 3).

**Table 3.** Incidence, Scaled Prevalence Rates, and Total Prevalence of Scar Malposition Recorded Between the Smooth Implant, Micro-Textured Implant, and Smooth Implant + Mesh Groups Throughout the Duration of Follow-up

Implant type	Minor malposition (1 < x ≤ 2 mm)	Moderate malposition (2 < x ≤ 3 mm)	Major malposition (>3 mm)	Total	Prevalence by cohort
Smooth implants (n = 84)	2	7	4	13	15.40%
Micro-textured implants (n = 49)	3	1	0	4	8.16%
Smooth implants + mesh (n = 67)	2	0	1	3	4.48%
Total	7	8	5	20	10.00%

The smooth silicone gel breast augmentation group with the prophylactic placement of PDO internal support matrix had a scar malposition rate of 4.48% and revealed a statistically significant difference in scar malposition rate compared with the smooth devices alone (*P*-value 0.03; 95% CI −0.21 to −0.01). There was no statistically significant difference in scar malposition rate between the micro-textured devices alone and smooth with mesh group (*P*-value 0.41; 95% CI −0.13 to 0.05). Of the 2 patients from the smooth implants with mesh group who developed scar malposition during the course of longitudinal follow-up, only 1 instance was bilateral, and the 2 non-bilateral cases fell under the minor malposition category, showing a trend toward decreased severity of scar malposition in affected patients with PDO mesh.

## Discussion

After the Allergan recall of macro-textured implants in the United States, only micro-textured devices have remained for textured options; looking back at the micro-textured vs smooth experience, there was little benefit to be seen to using micro-texture except in cases where shaped devices were needed. In this study, the authors sought to find an alternative to micro-textured devices in a smooth environment with greater pocket control. Due to the observed soft tissue support over the first 3 to 4 months of capsular maturation and favorable longitudinal results, the prophylactic placement of PDO mesh was determined to be a safe and viable alternative to micro-textured implants in preventing scar malposition and promoting more durable and predictable results. This study looked at patient results no longer than 2 years old and thus did not record any cases of BIA-ALCL or capsular contracture as these conditions typically develop in greater frequency over longer durations of time. The main purpose of this study was to evaluate the protective benefit of PDO mesh against scar malposition rather than investigate the development of these implant-related complications which have been evaluated on a larger level by other groups.

**Figure 2.**
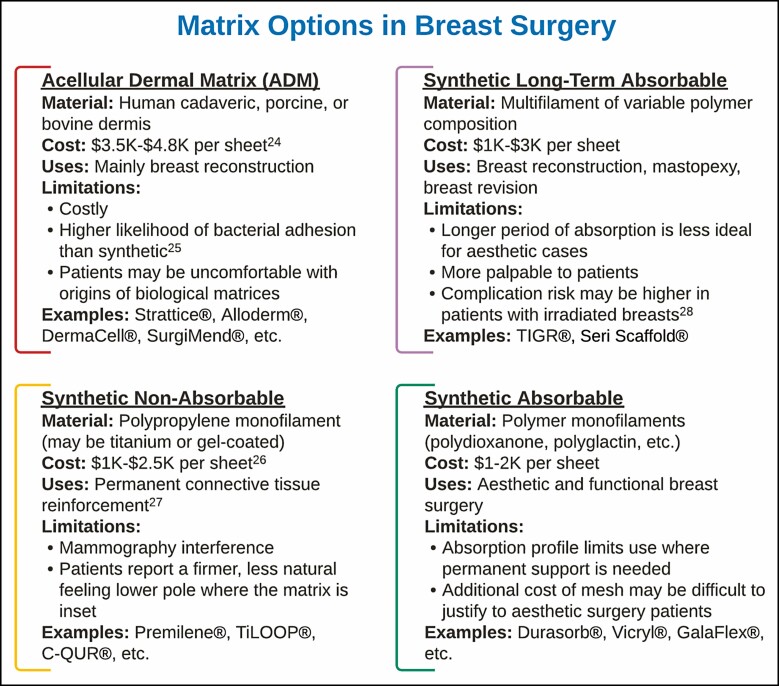
A summary of the material, monetary cost, benefit, and specific limitation profiles of the 4 most commonly used mesh categories in reconstructive and cosmetic breast surgery. “Cost” represents an estimated range based on current market prices and may vary by region and manufacturer.

Cost effectiveness is an important consideration when evaluating mesh use^15,16^ in self-paying cosmetic breast surgery, especially as a prophylactic measure. A variety of matrix options currently exist on the market and have been utilized in breast surgeries (Figure 2), albeit more commonly in a reconstructive rather than aesthetic context.^13^ These meshes include acellular matrices of bovine, porcine, or human cadaveric origin; permanent synthetic polymer options exist such as polypropylene; and a variety of absorbable synthetic polymers such as 4-hydroxybutyric acid.^17,18^ Accordingly, there is also a larger variation in the cost of these meshes, with synthetic polymers remaining the most inexpensive option, whereas biological meshes add thousands of US dollars to the cost of surgery.^19^ Due to the high cost of most mesh products, many plastic surgeons have largely avoided using them unless a patient’s revision or reconstructive case absolutely necessitated soft tissue supplementation.^20^ Use in primary augmentation mammoplasties is relatively novel and has not yet been described.

DuraSorb, a PDO polymer mesh by SIA Health, recently became available for use in aesthetic surgery and was originally considered for use in this study due to its clinical profile and low cost (DuraSorb mesh added a cost of approximately <$2000 USD to each bilateral augmentation case depending on patient needs and added 5-15 minutes of extra operating room/anesthesia time.). This matrix integrates into the surrounding soft tissue within 4 weeks, fully transfers load-bearing responsibility to native tissue within 3 months, and continues to stimulate local collagen production in its place until its full absorption at 1 year, leaving behind 1-2 mm of neo collagen vascular tissue in its place.^11-13^ DuraSorb, with its slightly shorter absorption profile of 3-12 months compared with that of P4HB (12-18 months) and other synthetics, was preferred for primary breast augmentation due to its unique combination of being a thin, non-palpable product with strength providing extra pocket control during the initial phase of collagen formation in wound healing. PDO mesh was judged to add minimal risk as an additional implanted device due to its composition; its similarity to the PDS (Ethicon, Inc., Raritan, NJ) suture renders it suitable for use in most patients.^21,22^ Previous work using P4HB against an implant in a small series of cases supported our assessment that mesh was sufficiently inert for device-on-device use.^23^ Different matrix options come with different drawbacks and benefits for use in breast surgery.^24-28^

**Figure 3.**
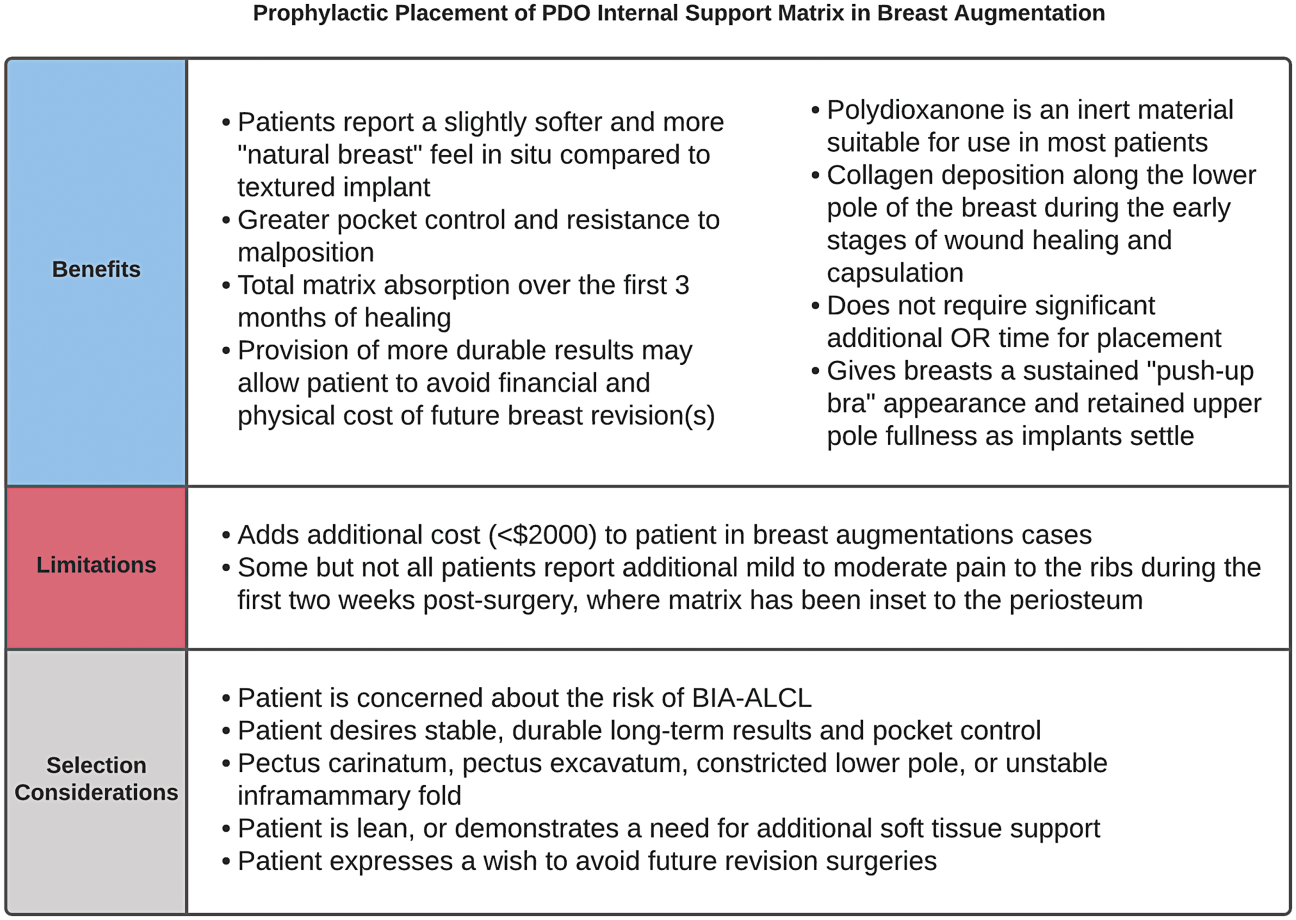
A summary of the benefits, limitations, and selection considerations for prophylactic placement of polydioxanone (PDO) internal support matrix in primary breast augmentation. BIA-ALCL, breast implant-associated anaplastic large cell lymphoma.

**Figure 4.**
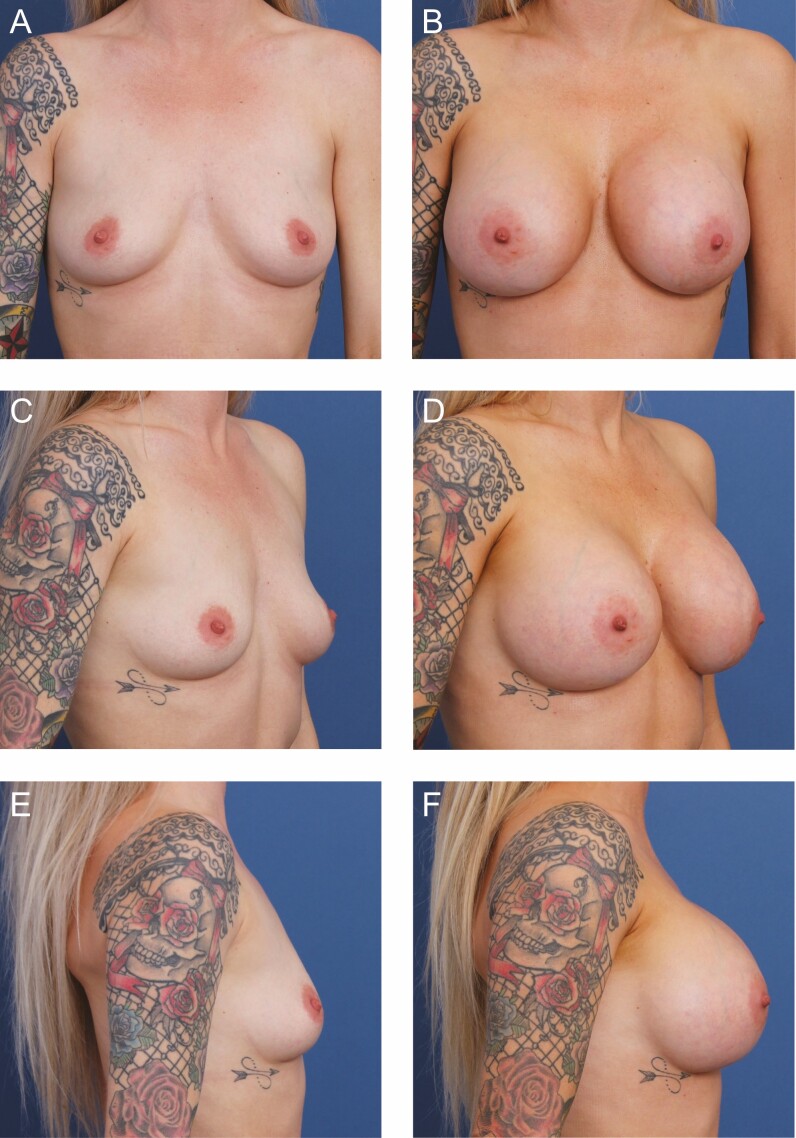
A 26-year-old female patient is shown 11 months after primary breast augmentation using Allergan Natrelle Inspira SoftTouch SSF Smooth Round Implants (Irvine, CA), 450 cc, plus DuraSorb (SIA, Chicago, IL) mesh. Mesh prophylaxis was employed for this patient based on the weak soft tissue at both inframammary folds. (A) Frontal, (C) three-quarter, and (E) lateral views are shown preoperatively; and (B) frontal, (D) three-quarter, and (F) lateral views are shown at a 9-month follow-up.

**Figure 5.**
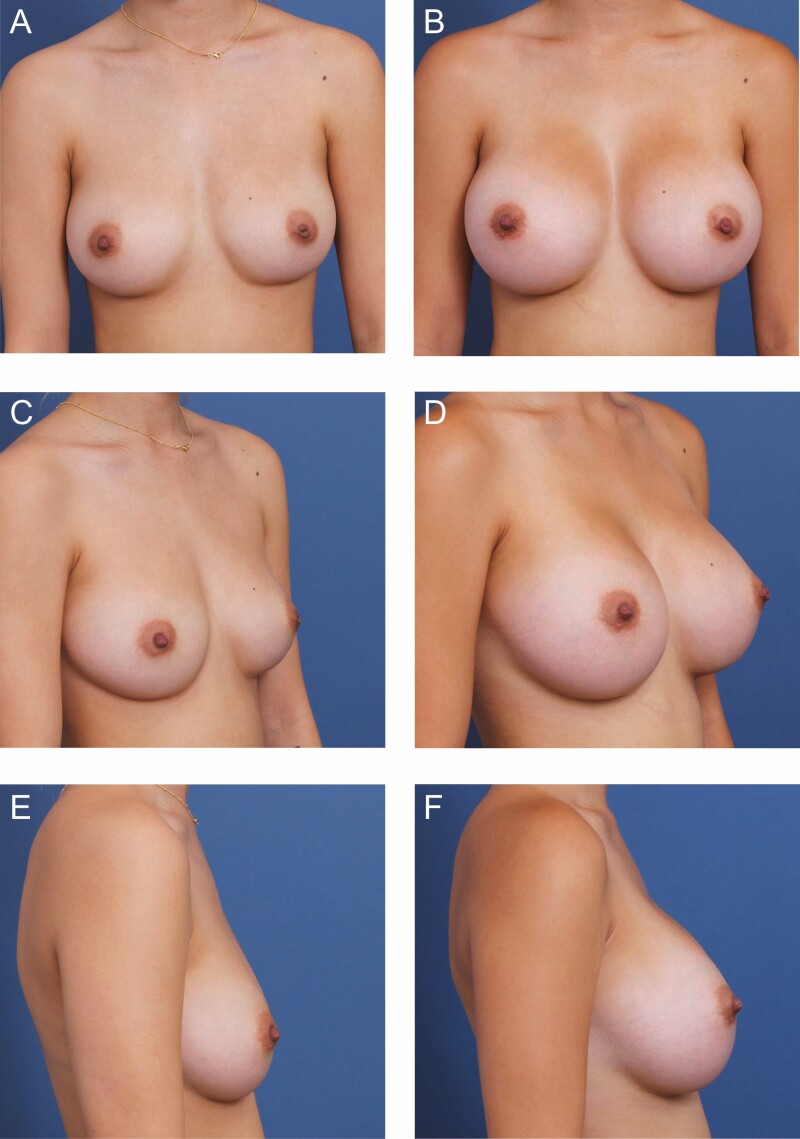
A 22-year-old female patient is shown 9 months after primary breast augmentation using Allergan Natrelle Inspira SoftTouch SSF Smooth Round Implants (Irvine, CA) 365 cc, plus DuraSorb (SIA, Chicago, IL) mesh. Due to the patient’s native lower pole volume and desire to undergo a proportionally high-volume augmentation, mesh prophylaxis was used to stabilize the lower poles to prevent bottoming out. (A) Frontal, (C) three-quarter, and (E) lateral views are shown preoperatively; and (B) frontal, (D) three-quarter, and (F) lateral views are shown at a 6-month follow-up.

**Figure 6.**
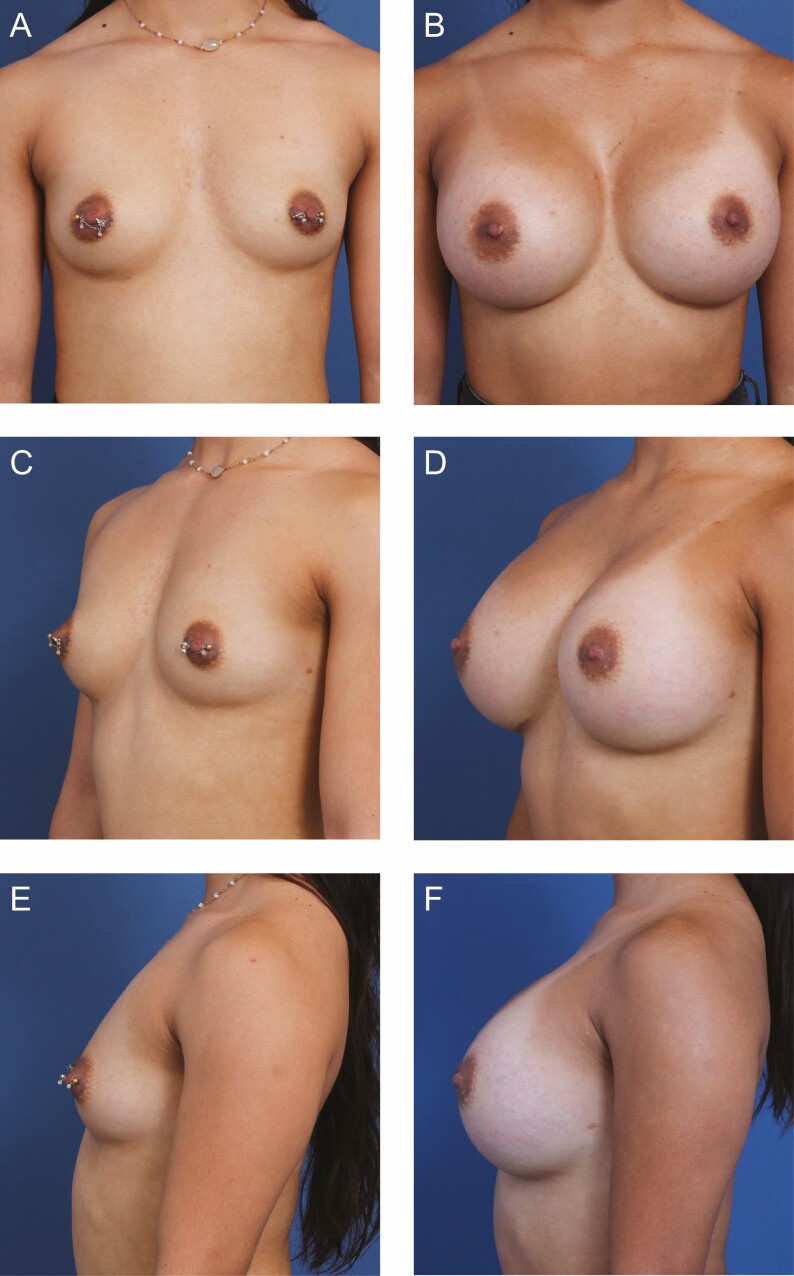
A 24-year-old female patient is shown 7 months after primary breast augmentation using Sientra HSC + High Profile Round Smooth Implants (Santa Barbara, CA), 505 cc, plus DuraSorb (SIA, Chicago, IL) mesh. Mesh was added to stabilize the inframammary fold as it was lowered and provide extra internal support for high-volume implants. (A) Frontal, (C) three-quarter, and (E) lateral views are shown preoperatively; and (B) frontal, (D) three-quarter, and (F) lateral views are shown at a 7-month follow-up.

**Figure 7.**
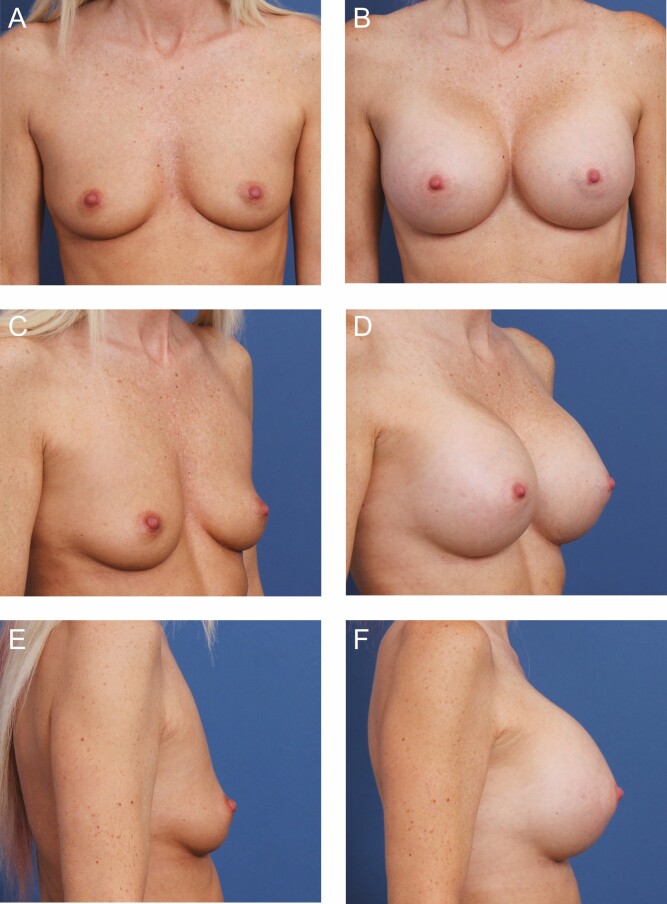
A 47-year-old female patient is shown 12 months after primary breast augmentation using Sientra HSC + High Profile Round Smooth Implants (Santa Barbara, CA), 470 cc, plus DuraSorb (SIA, Chicago, IL) mesh. This patient presented with thin, elastic soft tissue with depleted volume after breastfeeding. Mesh was used for bilateral pocket control and to give the patient sustained upper pole volume that she desired. (A) Frontal, (C) three-quarter, and (E) lateral views are shown preoperatively; and (B) frontal, (D) three-quarter, and (F) lateral views are shown at a 7-month follow-up.

**Figure 8.**
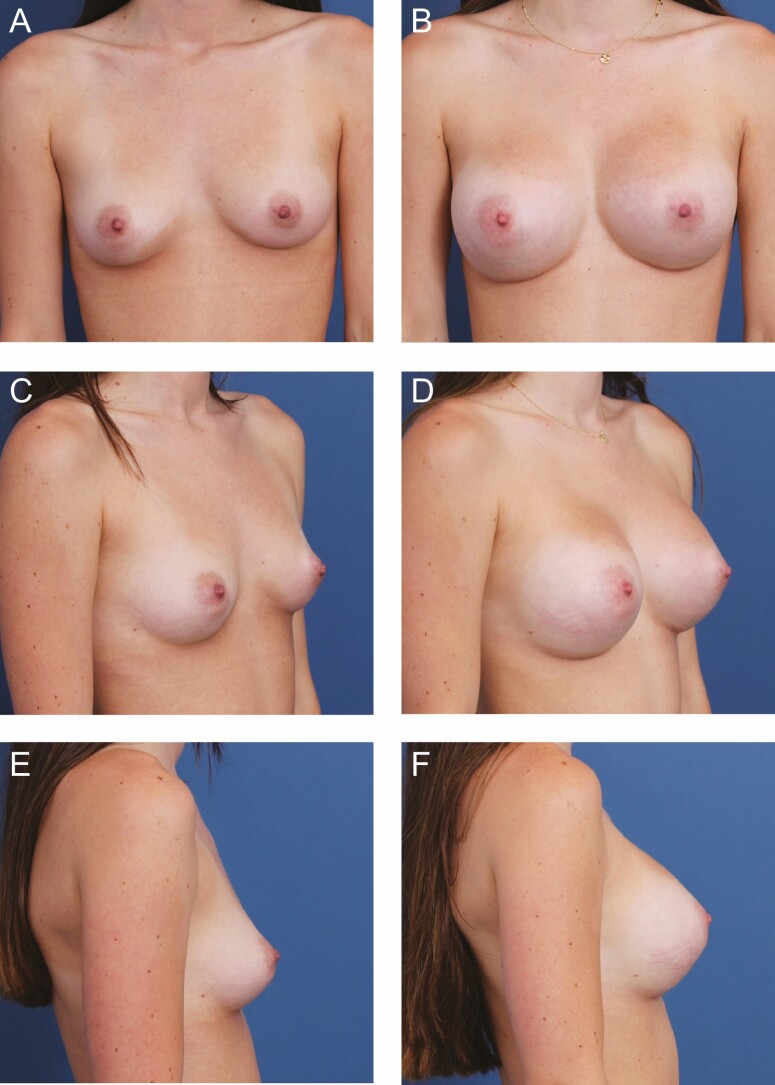
A 27-year-old female patient is shown 8 months after primary breast augmentation using Sientra HSC + High Profile Round Smooth Implants (Santa Barbara, CA), 385 cc, plus DuraSorb (SIA, Chicago, IL) mesh. Mesh was used on this patient to support a lowered inframammary fold to correct bilateral constricted lower poles. (A) Frontal, (C) three-quarter, and (E) lateral views are shown preoperatively; and (B) frontal, (D) three-quarter, and (F) lateral views are shown at a 7-month follow-up.

Certain patients present with anatomical features that necessitate careful implant selection to obtain good aesthetic results in a primary breast augmentation. Patients with an indiscrete IMF or one that needs to be lowered may require a micro-textured implant; such implants and/or shaped breast implants were used often by the senior author in patients with features of pectus carinatum, pectus excavatum, or a constricted lower pole as a “simple” breast augmentation on these patients may result in sub-optimal results without extra pocket control. For the patients who seem to fit a more “straight-forward breast augmentation” category and who lack any contraindications, mesh provides an extra layer of pocket support that may be protective and can minimize the risk of a later pocket revision or mastopexy by allowing greater precision, predictability, and durability of primary breast augmentation results (Figure 3). Though all patients in this cohort underwent dual-plane augmentation due to the primary surgeon’s aesthetic preference and the literature indicating a lower risk of capsular contracture with this method, the authors would have still elected to use mesh for stabilization of the implant if a subglandular or subfascial pocket had been used. Taken together, these benefits may be an especially important pull factor for patients wishing to avoid subsequent breast surgeries after their primary augmentation.^29,30^ More importantly, many patients seeking quality work demand greater precision and care and want as predictable of results as possible with minimal scar visibility. With a sufficiently affordable and inert option now on the market, mesh may serve its role in breast implant surgery as more than just a structural support or bail-out option in difficult revisional cases. These results serve as a promising indication of mesh’s potential as a literal safety net in patients undergoing aesthetic breast surgery. With the local and global market trending toward less use of textured breast implants, greater pocket control using smooth devices further stabilized with PDO mesh allows for superior patient outcomes with decreased risks associated with micro-textured implants (Figures 4-8).

## Conclusions

In silicone gel breast augmentation, micro-textured devices by themselves show a trend toward decreased scar malposition, although not statistically significant.^8^ This cohort study found that patients at high risk for scar migration with micro-textured breast implants give similar results to patients at average risk for malposition with smooth implants and must also be counseled by their physician on the risk of BIA-ALCL that accompanies their implants. For practitioners wishing to avoid this minimal risk altogether, as well as the conversation and controversy surrounding it, a viable alternative may include the use of mesh. The use of round, smooth silicone gel implants with the prophylactic placement of PDO internal support matrix was found to show a statistically significant trend toward decreased scar malposition compared with smooth implants alone. This protective effect may be beneficial for patients with anatomical features placing them at particularly high risk for malposition as well as patients seeking durable and long-term results from their primary breast augmentation. Patients who pay cash for an elective procedure seeking high-end plastic surgery from a board-certified plastic surgeon demand more precision than what may be considered the “norm,” and we sought to explore options to provide them with greater pocket control and precise results while optimizing safety and value. We, therefore, conclude that the prophylactic use of PDO internal support matrix in silicone gel breast augmentation is safe and has the lowest incidence of implant malposition, and also serves as a viable alternative to micro-textured breast implants.
